# Willingness to use and distribute HIV self-testing kits among people who inject drugs in the San Diego–Tijuana border region

**DOI:** 10.1186/s12954-023-00922-7

**Published:** 2024-01-03

**Authors:** Heather A. Pines, William H. Eger, Britt Skaathun, Carlos F. Vera, Alicia Harvey-Vera, Gudelia Rangel, Steffanie A. Strathdee, Angela R. Bazzi

**Affiliations:** 1 Division of Epidemiology and Biostatistics, School of Public Health, San Diego State University, San Diego, CA USA; 2 Herbert Wertheim School of Public Health and Human Longevity Science, University of California, San Diego, La Jolla, CA USA; 3 Department of Medicine, University of California, San Diego, La Jolla, CA USA; 4 School of Social Work, San Diego State University, San Diego, CA USA; 5 Mexico Section of the US-Mexico Border Health Commission, Tijuana, Baja California Mexico; 6 El Colegio de la Frontera Norte, Tijuana, Baja California Mexico; 7 School of Public Health, Boston University, Boston, MA USA

**Keywords:** HIV self-testing, Secondary distribution, Social networks, People who inject drugs, HIV prevention

## Abstract

**Background:**

HIV self-testing (HIVST) could increase HIV testing access among people who inject drugs (PWID), and secondary distribution (i.e., peer-delivery) of HIVST kits in PWID social networks could further expand coverage. We assessed willingness to use and distribute HIVST kits among PWID in the San Diego–Tijuana border region.

**Methods:**

From 2020 to 2021, HIV-negative PWID in San Diego, USA, and Tijuana, Mexico, completed surveys and provided data on individual (*N* = 539) and social network (*N* = 366) characteristics. We used modified Poisson regression to examine the effects of individual and social network characteristics on willingness to use and distribute HIVST kits.

**Results:**

Most participants were willing to use (81%) and distribute (81%) HIVST kits. At the individual level, prior HIV testing was positively associated with willingness to use (adjusted prevalence ratio [aPR] = 1.24, 95% confidence interval [CI] 1.10–1.40) and distribute (aPR = 1.27, 95% CI 1.12–1.43) HIVST kits, while perceiving oneself to be at higher HIV risk than others was negatively associated with willingness to use HIVST kits (aPR = 0.83, 95% CI 0.74–0.93). At the network level, willingness to distribute HIVST kits was positively associated with network size (aPR = 1.04 per member, 95% CI 1.01–1.08) and greater proportions of one’s network encouraging them to use drugs (aPR = 1.29, 95% CI 1.16–1.44) and having a history of homelessness (aPR = 1.51, 95% CI 1.31–1.74) or detention/arrest (aPR = 1.57, 95% CI 1.36–1.82), and negatively associated with a greater proportion of one’s network including “very close” persons (aPR = 0.80, 95% CI 0.69–0.94).

**Conclusions:**

We found high potential for HIVST kits and their secondary distribution to increase HIV testing among PWID who face the greatest barriers to facility-based testing.

## Background

People who inject drugs (PWID) remain disproportionately affected by human immunodeficiency virus (HIV) globally, with HIV risk estimated to be 35 times that among non-PWID [1]. In the USA, increasing rates of opioid and stimulant use are creating a volatile HIV risk environment for PWID [2–4]. At the same time, utilization of HIV prevention services among PWID, including HIV testing, remains suboptimal [5–8] and may have been further reduced during the COVID-19 pandemic [9, 10]. PWID face multilevel barriers to HIV testing, including HIV- [11, 12] and addiction-related [13, 14] stigma in healthcare settings and limited healthcare access due to homelessness, criminal justice involvement, and other structural factors [15]. As the ongoing opioid and polysubstance use epidemics compound the already complex challenges to HIV prevention among PWID [16], innovative strategies are needed to increase HIV testing for this population [17]. 

HIV self-testing (HIVST) enables discreet, convenient testing outside of healthcare settings, which could help circumvent many of the barriers to facility-based HIV testing among PWID [18, 19]. Recommended by the World Health Organization since 2016, HIVST is reliable [20], acceptable [18], and effective for increasing testing uptake and frequency among men who have sex with men (MSM), transgender persons, female sex workers (FSWs), young adults, and partners of pregnant and postpartum women and persons living with HIV [21–23]. While mail and facility-based delivery of HIVST kits are common, social network-based approaches (i.e., secondary distribution) of HIVST kits have shown promise for improving the reach of HIVST into marginalized populations with limited healthcare access [21]. For example, secondary distribution of HIVST kits has led to increased HIV testing and diagnoses among individuals who were never or infrequently tested for HIV, including the primary partners of pregnant women [24–26], sexual partners of FSWs [27, 28], and social network members of MSM [29]. 

While HIVST remains understudied among PWID, emerging evidence suggests that HIVST kits may be acceptable in this population, particularly when coupled with harm reduction services [30, 31]. Social network-based intervention strategies involving peer-driven outreach and education have been shown to reduce HIV risk [32–35] and increase facility-based HIV testing [36, 37] among PWID, and secondary distribution of sterile syringes and naloxone has been linked to reduced syringe sharing, HIV transmission risk, and overdose deaths [38–41]. Collectively, this work highlights the HIV prevention potential of HIVST kits for PWID, particularly if delivered via secondary distribution by peers.

We examined willingness to use and distribute HIVST kits among PWID in the San Diego–Tijuana border region, where HIV epidemics have been shaped by socio-contextual factors (e.g., poverty, migration, deportation, stigma, criminalization of PWID and sex workers) that heighten HIV risk while simultaneously limiting access to HIV prevention and care [42–44]. This region is also situated along a major drug trafficking corridor that has contributed to historically high rates of heroin and methamphetamine use in local communities [45, 46]. The relatively recent introduction of fentanyl into this drug supply presents new challenges [47], as fentanyl has been linked to increased injection frequency and receptive syringe sharing among PWID [48–55]. Furthermore, the international San Diego–Tijuana border crossing is one of the busiest land border crossings in the world, with studies documenting substantial cross-border mobility and drug use among PWID [56, 57]. Phylogenetic analyses of HIV-1 *pol* sequences from people living with HIV in this region also provide evidence of cross-border transmission [58–60], which threatens efforts to end the HIV epidemic in both countries. If network-based strategies promoting the secondary distribution of HIVST kits are acceptable among PWID along the San Diego–Tijuana border, interventions could leverage this approach in San Diego, Tijuana, and beyond.

## Methods

### Study population and design

We used baseline data from the prospective *La Frontera* study of trends in the incidence of HIV, hepatitis C virus (HCV), and drug-related overdose associated with binational drug markets and drug tourism in the San Diego–Tijuana border region (*n* = 612). As previously described [61], recruitment involved street outreach from mobile vans between October 2020 and October 2021 with the goal of sampling (1) ~ 200 San Diego residents who had crossed the border to inject drugs in Tijuana in the last 2 years, (2) ~ 200 San Diego residents who had not crossed the border to use drugs in Tijuana in the last 2 years, and (3) ~ 200 Tijuana residents who had not crossed the border to use drugs in the USA in the last 2 years. Eligibility criteria included: (1) ≥ 18 years of age, (2) injection drug use in the past month, and (3) being a San Diego or Tijuana resident. All study materials were available in English and Spanish and translated by binational research staff. After providing written informed consent, participants completed an interviewer-administered survey in English or Spanish, depending on their language preferences, via computer-assisted personal interviewing. Next, participants provided a fingerstick blood sample for rapid HIV and HCV testing (Miriad ® HIV/HCV Antibody InTec Rapid Anti-HCV Test; Avantor, Radnor, PA) and received their rapid test results and post-test counseling. Those with reactive or indeterminate results provided another fingerstick blood sample for a second HIV and/or HCV rapid test (Oraquick® HIV and Oraquick® HCV; Orasure, Bethlehem, PA). In the event of a second reactive result, participants provided blood samples for confirmatory testing at the San Diego Center for AIDS Research and were referred to local clinics for additional testing and healthcare follow-up. To reduce participant burden, two weeks after baseline visits, participants returned to complete supplemental surveys eliciting information on their social networks and HIVST. Participants received $20 each for completing baseline and supplemental survey visits, respectively. Institutional review boards at the University of California, San Diego and Xochicalco University approved all study procedures.

### Data collection

#### Outcomes of interest: willingness to use and distribute HIVST kits

Prior to asking participants about HIVST, interviewers introduced it to them with the following text: “*An HIV self-test is a rapid HIV test that you can give yourself at home or any convenient location by collecting your own saliva or blood via a finger prick with a small needle. If you test HIV-positive on an HIV self-test, you will still need to take a standard HIV test at a clinic or another community-based organization that performs HIV testing to confirm your test result*." Willingness to use HIVST kits was then assessed by asking participants, *“Would you be willing to use an HIV self-test to test yourself for HIV infection?”* with the following response options: “*definitely not,” “probably not,” “not sure or do not know,” “probably*,” or “*definitely*.” Those who responded “*definitely”* or “*probably”* were classified as willing to use HIVST kits. To further characterize perceptions of HIVST, participants who were willing to use HIVST kits were asked how much they agreed with reasons for motivation to use HIVST kits (e.g., “*I would be able to test for HIV more regularly”*) with 5-point Likert-scale response options (e.g., *“strongly disagree”* to *“strongly agree”*). Those who were unwilling to use HIVST kits were asked how much they agreed with reasons why some people might not want to use HIVST kits (e.g., “*I would be worried that HIV self-tests are less accurate than standard HIV tests”*) with the same 5-point Likert-scale response options. Willingness to distribute HIVST kits was assessed via two questions: *“Would you be willing to give your sexual partners HIV self-tests to test themselves for HIV infection?”* and *“Would you be willing to give your drug use partners HIV self-tests to test themselves for HIV infection?”* Response options included: *“definitely not,” “probably not,” “not sure or do not know,” “probably,”* or *“definitely*.” Those who responded “*definitely”* or “*probably”* to either question were classified as willing to distribute HIVST kits.

#### Exposures of interest: individual and social network characteristics

*Individual characteristics* included socio-demographics (age in years, sex assigned at birth [male or female], ethnicity [Hispanic/Latinx/Mexican or Non-Hispanic/Latinx/Mexican], years of education completed, homelessness in the past six months), HIV testing history (*“Have you ever been tested for HIV/AIDS before today?”*), hazardous alcohol consumption (Alcohol Use Disorders Identification Test [AUDIT-C] score ≥ 4 for men and ≥ 3 for women) [62], polydrug use (using ≥ 2 of the following in the past six months: heroin, crack cocaine, methamphetamine, fentanyl, PCP/Angel Dust, ecstasy), past six-month injection behaviors (*“How often did you inject (shoot) any drug or drug combinations?”*; *“How often did you use a syringe that you knew or suspected that it had been used before by someone else?”*), past six-month sexual behaviors (number of sexual partners; transactional sex [i.e., received something you needed, such as money, drugs, alcohol, shelter, food transportation, or protection, in exchange for sex]; alcohol or drug use before or during sex), perceived HIV risk (*“Compared to other drug users in this city, how likely do you think you are to get (infected with) HIV/AIDS?”*), and HIV pre-exposure prophylaxis (PrEP) awareness (*“Before today, had you ever heard of HIV-negative people taking HIV medications or PrEP before being exposed to HIV to protect against HIV infection?”*). PrEP interest was assessed by asking participants, *“What kind of PrEP product would you be interested in using?”* Check all that apply response options included: *“a pill I needed to take every day,” “an injection I needed to get every 2 months,” “a vaginal gel I needed to use before sex”* [women only], and *“none.”* Those who indicated interest in any modality were classified as interested in PrEP.

*Social network characteristics* were measured via a social network inventory that (1) assessed participants’ network size (*“Please tell me how many friends you associate with and talk to about things that are important to you that you have seen in the past 30 days”*), (2) generated a list of up to 20 members of participants’ networks (i.e., alters), starting with those who are closest to them, and (3) collected alter-specific information about the first five (i.e., closest) alters listed. Alter-specific measures included age in years, gender identity (male, female, transgender male, transgender female, or non-binary, genderqueer, gender fluid, or agender), relationship duration in years, closeness (*“How close do you feel to [alter]?”;* response options: “*very close,” “somewhat close,” “not very close,”* or *“not close at all”*), place of residence (*“Where does [alter] currently live?”;* response options: “*San Diego County*,” “*Tijuana*,” “*other*,” or “*don’t know*”), history of homelessness (*“Has [alter] ever lived on the street, in a shelter, in a single room occupancy hotel, temporarily with friends/relatives, in a car, or squatted?”*), history of detention or arrest (*“Has [alter] ever been detained or arrested?”*), sexual (vaginal or anal) relationship in the past six months, drug use frequency (*“How often does [alter] use drugs?”*; response options ranged from *“never”* to *“every day”*), injection drug use (*“Does [alter] use drugs by injection, by non-injection, or sometimes one and sometimes the other?”*), injection equipment sharing (*“Have you ever used a needle, water, cooker, or cotton that had already been used by [alter]?”*), drug sharing (*“Has [alter] ever offered to share or encouraged you to use drugs?”)*, and drug risk communication (*“Has [alter] ever encouraged you to stop using drugs?”* and *“Has [alter] ever encouraged you to specifically stop using drugs by injection?”*).

### Statistical analysis

We calculated descriptive statistics to characterize the sample overall and by willingness to use and distribute HIVST kits. We then modeled our outcomes of interest as a function of exposures hypothesized to influence each outcome using modified Poisson regression with robust error variance to estimate prevalence ratios (PRs) and corresponding 95% confidence intervals (CIs) for our exposure-outcome relationships of interest since logistic regression yields odds ratios that overestimate PRs when the outcome is not rare [63, 64]. We fit separate multivariable models for each exposure-outcome relationship of interest and identified covariates as sufficient for confounding control and inclusion in each model via directed acyclic graphs [65], which we constructed to depict hypothesized interrelationships among the exposure, outcome, and other relevant covariates for each exposure-outcome relationship of interest (see Tables 3 and 4). In sensitivity analyses, we examined willingness to distribute HIVST kits to sexual partners and to drug use partners as separate outcomes. We excluded participants who did not complete the supplemental survey (*n* = 26) and those who tested HIV positive or reported a prior HIV diagnosis in the supplemental survey (*n* = 47), since participants who reported a prior HIV diagnosis were not asked HIVST questions in the supplemental survey, for a total sample size of 539. In analyses of social network characteristics, we excluded participants who did not consent to completing the social network inventory (*n* = 95) and did not report at least one alter in the inventory (*n* = 78) for a final sample size of 366. We used SAS 9.4 (SAS Institute, Inc; Cary, NC) to conduct all analyses.

## Results

### Sample characteristics

Our total sample (*n* = 539) had a mean age of 43.3 years (standard deviation [SD] = 10.8), 75% were assigned male sex at birth, 72% identified as Hispanic/Latinx/Mexican, 69% lived in San Diego, and 46% reported homelessness in the past six months (Table 1). While 74% of participants reported prior HIV testing, only 34% reported testing in the past 12 months. Despite only 29% of participants perceiving themselves to be more likely to get HIV than other PWID, many reported past six-month polydrug use (78%), injecting drugs multiple times daily (69%), using alcohol or drugs before or during sex (52%), and using syringes they knew or suspected were used before (51%). Among participants who provided social network data (*n* = 366), their networks had a mean of 3.2 members (SD = 2.1), and on average networks consisted of mostly persons they described as male (71%), using drugs daily (65%), injecting drugs (58%), and having a history of homelessness (58%) and detention or arrest (56%). On average, participants reported being “very close” with 40% of their network members, having had sex with 13%, using injection equipment after 30%, and receiving encouragement to use drugs from 40%.

**Table 1:** Characteristics of HIV-negative PWID in the San Diego–Tijuana border region (*N* = 539)

Individual characteristics (*N* = 539)	*n*	%
Socio-demographics		
Age (in years)	Mean = 43.25	SD = 10.80
Identifies as Hispanic/Latinx/Mexican	389	72.2
Assigned male sex at birth	403	74.8
Years of education	Mean = 9.86	SD = 3.43
San Diego resident	370	68.7
Homeless (past 6 months)	249	46.2
HIV testing history		
Lifetime	396	73.9
Past 12 months	180	34.5
Substance use and injection behaviors (past 6 months)		
Hazardous alcohol consumption^a^	103	19.1
Polydrug use^b^	414	78.0
Injected drugs multiple times daily	370	68.7
Used a syringe that you knew/suspected had been used before	277	51.4
Sexual behaviors (past 6 months)		
Number of sexual partners	Mean = 5.1	SD = 44.5
Any vaginal or anal intercourse	294	54.7
Any transactional sex^c^	64	11.9
Any alcohol or drug use before or during sex	281	52.2
Perceived risk of HIV		
More likely to get HIV than other PWID in this city	154	28.8
PrEP		
Previously aware of PrEP	94	17.4
Interested in using any type of PrEP (oral, injectable, or vaginal)	457	84.9

Social network characteristics (*N* = 366)	Mean	SD
Network size	3.22	2.13
Network age (in years)	40.32	10.14
Gender Identity		
Proportion of network that identifies as male	0.71	0.34
Proportion of network that identifies as female	0.28	0.33
Duration of network relationships (in years)	8.40	10.06
Closeness		
Proportion of network with whom they are very close	0.40	0.39
Place of residence		
Proportion of network that lives in San Diego	0.44	0.48
Proportion of network that lives in Tijuana	0.54	0.48
Housing		
Proportion of network that has ever been homeless^d^	0.58	0.44
History of detention or arrest		
Proportion of network that has ever been detained or arrested	0.56	0.44
Sexual intercourse (past 6 months)		
Proportion of network with whom they had vaginal or anal intercourse	0.13	0.24
Drug use behaviors		
Proportion of network that uses drugs every day	0.65	0.41
Proportion of network that injects drugs	0.58	0.42
Proportion of network with whom they share injection equipment	0.30	0.40
Proportion of network that offered to share/encouraged them to use drugs	0.40	0.42
Drug risk communication		
Proportion of network that has ever encouraged them to stop using drugs	0.33	0.41
Proportion of network that has ever encouraged them to stop injecting drugs	0.31	0.40

*PrEP* Pre-exposure prophylaxis; *PWID* People who inject drugs; *SD* Standard deviation

^a^Alcohol Use Disorders Identification Test [AUDIT-C] score ≥ 4 for men and ≥ 3 for women

^b^Polydrug use = used ≥ 2 of the following drugs: heroin, cocaine, methamphetamine, fentanyl, PCP/angel dust, ecstasy

^c^Transactional sex = received something you needed (such as money, drugs, alcohol, shelter, food transportation, or protection) in exchange for sex

^d^Homeless = ever lived on the street, in a shelter, in a single room occupancy hotel, temporarily with friends/relatives, in a car, or squatted

### Willingness to use HIVST kits

Overall, 81% of participants were willing to use HIVST kits (Table 2). Among those willing to use HIVST kits (*n* = 436), most viewed HIVST kits as more private and confidential (95%) and convenient (91%) than standard, facility-based HIV testing, and believed that HIVST would enable them to test for HIV more regularly (94%). Among those unwilling to use HIVST kits (*n* = 103), nearly one quarter worried about HIVST kits’ accuracy (24%) and potential for incorrect use (24%) or misinterpretation of results (24%). Willingness to use HIVST kits (Table 3) was positively associated with more years of education (adjusted prevalence ratio [aPR] = 1.02 per year, 95% confidence interval [CI] 1.01–1.04), prior HIV testing (aPR = 1.24, 95% CI 1.10–1.40), and hazardous alcohol consumption (aPR = 1.12, 95% CI 1.04–1.22), while it was negatively associated with perceiving oneself to be more likely to get HIV than other PWID (aPR = 0.83, 95% CI 0.74–0.93), injecting drugs multiple times daily (past six months; aPR = 0.87, 95% CI 0.80–0.95), and using syringes known or suspected to have been used before (past six months; aPR = 0.92, 95% CI 0.85–1.00).

**Table 2:** Willingness to use and distribute HIVST kits among HIV-negative PWID in the San Diego–Tijuana border region (*N* = 539)

	*n*	%
Willing to use an HIV self-test	436	80.9
Willing to distribute HIV self-tests to sexual partners or drug use partners	437	81.1
Willing to give HIV self-tests to sexual partners	423	78.6
Willing to give HIV self-tests to drug use partners	406	75.3
I would not want to use an HIV self-test because… (*N* = 103)		
I would be worried that HIV self-tests are less accurate than standard HIV tests	25	24.3
I would be worried about using the test incorrectly	25	24.3
I would be worried about misinterpreting the test result	25	24.3
I would be motivated to use an HIV self-test because… (*N* = 436)		
I would be able to test for HIV more regularly	410	94.0
It would be more convenient than going to a clinic/CBO for a standard HIV test	396	90.8
It would give me more privacy and help ensure the confidentiality of my HIV test result	413	94.7

*CBO* Community-based organization, *HIVST* *HIV* Self-testing, *PWID* People who inject drugs

**Table 3:** Individual characteristics associated with willingness to use HIVST kits among HIV-negative PWID in the San Diego–Tijuana border region (*N* = 539)

	Willing to use HIVST kits		Adjusted	
	No (*N* = 103)	Yes (*N* = 436)		
	*n* (column %)	*n* (column %)	PR	95% CI
Socio-demographics				
Mean age in years	44.3 (SD = 10.2)	43.0 (SD = 10.9)	0.998	0.994, 1.001
Identifies as Hispanic/Latinx/Mexican	76 (73.8)	313 (71.8)	0.98	0.90, 1.07
Assigned male sex at birth	80 (77.7)	323 (74.1)	0.96	0.88, 1.06
San Diego resident	68 (66.0)	302 (69.3)	1.03	0.94, 1.13
Mean years of education^a^	8.8 (SD = 3.7)	10.1 (SD = 3.3)	1.02	1.01, 1.04
Homeless (past 6 months)^b^	41 (39.8)	208 (47.7)	1.06	0.97, 1.15
HIV testing history				
Lifetime^c^	57 (55.3)	339 (78.3)	1.24	1.10, 1.40
Past 12 months^c^	30 (29.7)	150 (35.5)	1.03	0.95, 1.12
Substance use and injection behaviors (past 6 months)				
Hazardous alcohol consumption^d,h^	9 (8.7)	94 (21.6)	1.12	1.04, 1.22
Polydrug use^d,i^	72 (70.6)	342 (79.7)	1.06	0.94, 1.19
Injected drugs multiple times daily^d^	82 (79.6)	288 (66.1)	0.87	0.80, 0.95
Used a syringe that you knew/suspected had been used before^d^	62 (60.2)	215 (49.3)	0.92	0.85, 1.00
Sexual behaviors (past 6 months)				
Mean number of sexual partners^e^	3.3 (SD = 12.7)	5.5 (SD = 49.0)	1.0000	0.9998, 1.0003
Any transactional sex^e,j^	6 (5.9)	58 (13.3)	1.09	0.97, 1.21
Any alcohol or drug use before or during sex^e^	48 (47.1)	233 (53.4)	1.01	0.93, 1.10
Perceived risk of HIV				
More likely to get HIV than other PWID in this city^f^	46 (44.7)	108 (25.1)	0.83	0.74, 0.93
PrEP				
Previously aware of PrEP^g^	11 (10.7)	83 (19.0)	1.04	0.95, 1.13
Interested in using any type of PrEP (oral, injectable, or vaginal)^g^	91 (88.4)	366 (84.1)	1.02	0.91, 1.14

*CI* Confidence interval; *HIVST* HIV self-testing; *PR* Prevalence ratio; *PrEP* Pre-exposure prophylaxis; *PWID* People who inject drugs; *SD* Standard deviation

^a^Adjusted for age, ethnicity, assigned sex at birth, and city of residence

^b^Adjusted for the covariates noted in (a) and education

^c^Adjusted for the covariates noted in (b) and homelessness

^d^Adjusted for the covariates noted in (c) and prior HIV testing

^e^Adjusted for the covariates noted in (d) and polydrug use

^f^Adjusted for the covariates noted in (e) and number of sexual partners

^g^Adjusted for the covariates noted in (f) and perceived risk of HIV

^h^Alcohol Use Disorders Identification Test [AUDIT-C] score ≥ 4 for men and ≥ 3 for women

^i^Polydrug use = used ≥ 2 of the following drugs: heroin, cocaine, methamphetamine, fentanyl, PCP/angel dust, ecstasy

^j^Transactional sex = received something you needed (such as money, drugs, alcohol, shelter, food transportation, or protection) in exchange for sex

### Willingness to distribute HIVST Kits

Overall, 81% of participants were willing to distribute HIVST kits to others (Table 2), including sexual partners (79%) or drug use partners (75%). At the individual level (Table 4), willingness to distribute HIVST kits was positively associated with more years of education (aPR = 1.02 per year, 95% CI = 1.01–1.04), prior HIV testing (aPR = 1.27, 95% CI 1.12–1.43), and willingness to use HIVST kits (aPR = 8.31, 95% CI 4.88–14.17). At the network level, willingness to distribute HIVST kits was positively associated with network size (aPR = 1.04 per member, 95% CI 1.01–1.08) and greater proportions of one’s network encouraging them to use drugs (aPR = 1.29, 95% CI 1.16–1.44) and having a history of homelessness (aPR = 1.51, 95% CI 1.31–1.74) or detention/arrest (aPR = 1.57, 95% CI 1.36–1.82). Willingness to distribute HIVST kits was lower among participants whose networks consisted of a greater proportion of persons they considered “very close” to them (aPR = 0.80, 95% CI 0.69–0.94). Results were qualitatively the same in models that considered willingness to distribute HIVST kits to sexual partners and to drug use partners as separate outcomes (data not shown).

**Table 4:** Individual and social network characteristics associated with willingness to distribute HIVST kits to sexual partners or drug use partners among HIV-negative PWID in the San Diego–Tijuana border region

	Willing to distribute HIVST kits				Adjusted	
	No (N = 102)		Yes (N = 437)			
Individual characteristics (N = 539)	*n*	(column %)	*n*	(column %)	PR	95% CI
Socio-demographics						
Mean age in years	44.7	SD = 10.1	42.9	SD = 11.0	0.997	0.994, 1.001
Identifies as Hispanic/Latinx/Mexican	78	76.5	311	71.2	0.95	0.87, 1.04
Assigned male sex at birth	78	76.5	325	74.4	0.98	0.89, 1.07
San Diego resident	62	60.8	308	70.5	1.09	0.99, 1.20
Mean years of education^a^	8.7	SD = 3.7	10.1	SD = 3.3	1.02	1.01, 1.04
Homeless (past 6 months)^b^	40	39.2	209	47.8	1.06	0.98, 1.15
HIV testing history						
Lifetime^c^	55	53.9	341	78.6	1.27	1.12, 1.43
Past 12 months^c^	30	29.7	150	35.5	1.03	0.95, 1.12
Willing to use HIVST kits^c^	11	10.8	425	97.3	8.31	4.88, 14.17

	Willing to distribute HIVST kits				Adjusted	
	No (*N* = 70)		Yes (*N* = 296)			
Social network characteristics (N = 366)	Mean	SD	Mean	SD	PR	95% CI
Network size^d^	1.94	0.93	3.52	2.22	1.04	1.01, 1.08
Network age (in years)^e^	40.00	12.45	40.40	9.52	1.00	0.99, 1.01
Gender Identity						
Proportion of network that identifies as male^e^	0.70	0.39	0.72	0.33	1.04	0.88, 1.22
Proportion of network that identifies as female^e^	0.30	0.39	0.27	0.32	0.94	0.80, 1.12
Duration of network relationships (in years)^e^	9.45	12.78	8.15	9.30	0.996	0.990, 1.002
Closeness						
Proportion of network with whom they are very close^e^	0.57	0.44	0.36	0.37	0.80	0.69, 0.94
Place of residence						
Proportion of network that lives in San Diego^e^	0.34	0.47	0.47	0.48	1.11	0.96, 1.28
Proportion of network that lives in Tijuana^e^	0.65	0.47	0.52	0.48	0.89	0.77, 1.03
Housing						
Proportion of network that has ever been homeless^e,f^	0.21	0.37	0.68	0.41	1.51	1.31, 1.74
History of detention or arrest						
Proportion of network that has ever been detained or arrested^e^	0.17	0.34	0.66	0.41	1.57	1.36, 1.82
Sexual intercourse (past 6 months)						
Proportion of network with whom they had vaginal or anal intercourse^e^	0.12	0.29	0.13	0.23	1.01	0.78, 1.31
Drug use behaviors						
Proportion of network that uses drugs every day^e^	0.69	0.44	0.64	0.40	0.92	0.81, 1.04
Proportion of network that injects drugs^e^	0.57	0.47	0.58	0.41	1.03	0.91, 1.17
Proportion of network with whom they share injection equipment^e^	0.25	0.40	0.31	0.40	1.08	0.95, 1.24
Proportion of network that offered to share/encouraged them to use drugs^e^	0.19	0.33	0.45	0.43	1.29	1.16, 1.44
Drug risk communication						
Proportion of network that has ever encouraged them to stop using drugs^e^	0.32	0.43	0.33	0.40	0.98	0.86, 1.12
Proportion of network that has ever encouraged them to stop injecting drugs^e^	0.31	0.43	0.32	0.40	0.98	0.86, 1.13

*CI* Confidence interval; *HIVST* HIV self-testing; *PR* Prevalence ratio; *PrEP* Pre-exposure prophylaxis; *PWID* People who inject drugs; *SD* Standard deviation

^a^Adjusted for age, ethnicity, assigned sex at birth, and city of residence

^b^Adjusted for the covariates noted in (a) and education

^c^Adjusted for the covariates noted in (b) and homelessness

^d^Adjusted for the covariates noted in (c), prior HIV testing, and polydrug use

^e^Adjusted for the covariates noted in (d) and network size

^f^Homeless = ever lived on the street, in a shelter, in a single room occupancy hotel, temporarily with friends/relatives, in a car, or squatted

## Discussion

We examined willingness to use and distribute HIVST kits to social network members among PWID in the San Diego–Tijuana border region. We found HIVST kits to be largely acceptable, with four out of five participants willing to use HIVST kits themselves. For most of these participants, privacy, convenience, and the potential for more regular HIV testing motivated willingness to use HIVST kits. Most of our sample also expressed a willingness to distribute HIVST kits to members of their social networks, including sexual and drug use partners. Given that PWID often have experience with the successful secondary distribution of syringes, naloxone, and other essential harm reduction supplies [38–41, 66, 67], our findings underscore the potential for HIVST kits and their secondary distribution (i.e., peer-delivery) to increase HIV testing in PWID social networks in this region and likely elsewhere.

Our findings are consistent with the high acceptability of HIVST documented in other HIV-affected populations [18] and emerging evidence that PWID view HIVST [30, 31] and other self-testing strategies (e.g., self-testing for HCV [HCVST]) [68–70] as acceptable and feasible. For example, a study of HIVST kits distributed from a Kentucky syringe service program (SSP) identified few usability problems and high interest in using HIVST kits every six months [31]. However, as noted by nearly a quarter of participants in our study who expressed unwillingness to use HIVST kits, concerns regarding test accuracy, ease of use, and interpretation of results may remain. Indeed, research on HCVST among PWID internationally has found that errors can occur during the testing process and that assistance may be needed while testing and interpreting results [68–70]. Additional challenges with HIVST technologies (e.g., HIVST kits that require individuals to mail samples for processing and call later for results) may also present barriers [30]. Thus, additional education on correct use and support throughout the testing process from experienced peers or SSP staff, for example, may facilitate successful uptake and interpretation of HIVST kits among PWID.

Our findings also suggest that additional supports may be needed to enhance the impact of HIVST kits for PWID facing the greatest social and structural challenges. We found that higher levels of education and prior HIV testing were positively associated with willingness to use HIVST kits, suggesting that messaging on the importance of HIV testing may help encourage PWID with lower levels of education and no HIV testing history to use HIVST kits. Additionally, while willingness to use HIVST kits was higher among participants who reported hazardous alcohol consumption, injecting drugs multiple times daily, and receptive syringe sharing, it was lower among participants who perceived themselves to be at higher HIV risk than other PWID. These findings imply that interventions to support the adoption of HIVST among PWID may need to be tailored to different individuals’ needs, possibly targeting a combination of HIV knowledge, risk perceptions, fear of learning one’s HIV status or facing HIV-related stigma, or motivation to engage in HIV testing, prevention, or treatment services due to competing priorities [71, 72]. 

When coupled with prior research on secondary syringe exchange [73, 74], our finding that most PWID in our sample were willing to distribute HIVST kits in their social networks suggests that secondary distribution of HIVST kits could be a powerful strategy for increasing HIV testing coverage in broader PWID communities. For example, research has found that secondary syringe exchange is likely helpful for reaching more marginalized populations of PWID who do not routinely access prevention services directly through SSPs or pharmacies [73, 74]. At the individual level, we found that participants with higher levels of education, prior HIV testing, and willingness to use HIVST kits themselves were more willing to distribute HIVST kits within their social networks. These findings align with recent research on PrEP use among PWID finding that, while most preferred keeping their PrEP use private, a minority expressed willingness to serve as PrEP education “champions” among their peers [75]. In our study, we also found that specific social network characteristics were associated with willingness to distribute HIVST kits (e.g., greater social network size, more network members who share drugs or encourage drug use, more network members with homelessness or detention or arrest experience). These findings suggest that secondary distribution may help reach more vulnerable PWID who face the greatest barriers to standard, facility-based HIV testing. However, willingness to distribute HIVST kits was lower for participants whose social networks consisted of more members with whom they are “very close,” suggesting that interventions may need to address fear of HIV-related stigma from close peers to support the secondary distribution of HIVST kits.

While our findings highlight the promise of HIVST kits and their secondary distribution for increasing HIV testing among PWID, testing is only one initial step to engaging individuals in HIV prevention and care. Additional research on how to best link PWID using HIVST kits to post-test counseling and HIV prevention (e.g., PrEP) and care (e.g., antiretroviral therapy [ART]) will be critical to harnessing the full potential of secondary HIVST kit distribution. While digital interventions have been developed to support linkage to HIV services following HIVST [76], PWID most vulnerable to HIV often lack access to mobile phones, the Internet, and other technologies that could support access to such interventions [77–79]. PWID often prefer to access health services, information, and referrals through SSPs [13, 72, 80, 81], which have a long history of deploying peers (i.e., “secondary exchangers”) in distributing harm reduction supplies within their social networks [38–41, 66, 67]. As such, social network-based strategies that leverage SSPs’ trusting relationships with PWID and success in promoting secondary distribution of prevention supplies could support increased HIV testing and subsequent engagement in HIV prevention and care.

Despite its many strengths, our study has several limitations. First, the geographic scope and unique context of this study, conducted in the San Diego–Tijuana border region, may limit the generalizability of our findings. However, this large cohort of PWID has experienced many of the socio-structural barriers to HIV testing and healthcare access (e.g., stigma, homelessness, criminalization of drug use) documented in other regions. Second, despite efforts to increase participants’ comfort providing information on their social networks (e.g., reminding them of the confidentiality and privacy protections in place), 32% of our sample did not report on their social networks. Third, our outcomes reflect hypothetical willingness to use and distribute HIVST kits, which may differ from actual use and distribution. To optimize the impact of HIVST among PWID, additional quantitative and qualitative research is needed to explore the concerns, experiences, and educational needs of individuals who receive HIVST kits.

## Conclusions

We found high levels of willingness to use and distribute HIVST kits among PWID in the San Diego–Tijuana border region, highlighting the potential for HIVST kits to increase HIV testing within PWID communities. Efforts to leverage effective secondary distribution and social network-based strategies within SSPs to support correct HIVST kit use, bolster HIV knowledge, address fears of HIV-related stigma, and facilitate linkage to HIV prevention and care may enhance the impact of HIVST kits and their secondary distribution among PWID.

## Data Availability

The datasets generated and/or analyzed during the current study are not publicly available but are available upon reasonable request to the Principal Investigator of *La Frontera*, Dr. Steffanie Strathdee (sstrathdee@health.ucsd.edu).

## Abbreviations

aPR: Adjusted prevalence ratio
ART: Antiretroviral therapy
AUDIT-C: Alcohol use disorders identification test
CI: Confidence interval
HCV: Hepatitis C virus
HIV: Human immunodeficiency virus
HIVST: HIV self-testing
FSWs: Female sex workers
MSM: Men who have sex with men
PrEP: Pre-exposure prophylaxis
PWID: People who inject drugs
SD: Standard deviation
SSP: Syringe service program
USA: United States of America
